# Xenobiology: A new form of life as the ultimate biosafety tool

**DOI:** 10.1002/bies.200900147

**Published:** 2010-04

**Authors:** Markus Schmidt

**Affiliations:** Organisation for International Dialogue and Conflict ManagementKaiserstr. 50/6, 1070 Vienna, Austria

**Keywords:** auxotrophy, biosafety, synthetic biology, xenobiology, xeno nucleic acids

## Abstract

Synthetic biologists try to engineer useful biological systems that do not exist in nature. One of their goals is to design an orthogonal chromosome different from DNA and RNA, termed XNA for xeno nucleic acids. XNA exhibits a variety of structural chemical changes relative to its natural counterparts. These changes make this novel information-storing biopolymer “invisible” to natural biological systems. The lack of cognition to the natural world, however, is seen as an opportunity to implement a genetic firewall that impedes exchange of genetic information with the natural world, which means it could be the ultimate biosafety tool. Here I discuss, why it is necessary to go ahead designing xenobiological systems like XNA and its XNA binding proteins; what the biosafety specifications should look like for this genetic enclave; which steps should be carried out to boot up the first XNA life form; and what it means for the society at large.

The best way to predict the future is to create it.

Peter Drucker

It is when we all play safe that we create a world of utmost insecurity.

Dag Hammerskjöld

## Introduction

In schools all over the world students learn that the secrets of life are encoded in the DNA molecule. Mainstream science is a true believer in DNA as the only stable genetic information storage, and understanding and modifying this monopolistic biopolymer has become the ultimate goal in contemporary bio-based R&D. Some scientists, however, have started to search for alternatives. They belong to apparently very different science fields and their quest for biochemical diversity is driven by different motivations.^1^–^3^ The science fields in question include four areas: origin of life, exobiology, systems chemistry, and synthetic biology (SB). The ancient Greeks, including Aristotle, believed in *Generatio spontanea*, the idea that life could suddenly come into being from non-living matter on an every day basis. Spontaneous generation of life, however, was finally discarded by the scientific experiments of Pasteur, whose empirical results showed that modern organisms do not spontaneously arise in nature from non-living matter. On the sterile earth 4 billion years ago, however, abiogenesis must have happened at least once, eventually leading to the last universal common ancestor (LUCA). LUCA's genetic code must have been based on DNA with four bases that form the three-nucleotide codons coding for 20 amino acids.^4^,^5^ The origin of life community tries to understand the processes of abiogenesis that caused LUCA (and all known life forms on earth) to use exactly this chemistry and this code to store genetic information. Why did it happen this way and not another? Some researchers have even proposed the idea that there could also be other more exotic life forms. Such postulated weird life could be a remnant of a different (earlier or even later) abiogenesis on earth. If it were to exist, it could be distinguished by its reliance on different chemical processes, biochemical building blocks, codes, or metabolism. In contrast to the earth-bound origin of life community, astrobiologists search for (unusual) life forms beyond Earth. Many people will have heard media reports about the Search for Extra-Terrestrial Intelligence (SETI) in the universe: the search for signals from extra-terrestrial life forms capable of sending them. Meanwhile, there is another less-known aspect of astrobiology. In this second field of activity, called exobiology, the aim is to search the solar system for evidence of non-intelligent life forms (such as microbes). On some celestial bodies “alien” life forms may have developed, say through the use of a solvent other than water or the use of very different chemical elements, *e.g*., silicon rather than carbon.^6^ Of course, there could also be other possibilities such as variations in the tripartite DNA-RNA-protein architecture found in earth life forms.^7^ Another research field that explores unnatural biochemical systems or biological subsystems is systems chemistry, that includes research on chemical self-organization, self-replicating, and self-reproducing chemical systems.^8^,^9^ While systems chemistry looks at the chemical level, SB is the design and construction of new biological systems not found in nature. SB aims at creating novel organisms for practical purposes, but also at gaining insights into living systems by re-constructing them. SB is developing rapidly as a new interdisciplinary field, involving microbiology, genetic engineering, information technology, nanotechnology, and biochemistry. SB as a scientific and engineering field includes the following subfields:^3^,^10^–^12^

- Engineering DNA-based biological circuits, including but not limited to standardized biological parts;
- Defining a minimal genome/minimal life (top-down approach);
- Constructing so-called protocells, *i.e*., living cells, from scratch (bottom-up approach);
- Production of gene fragments and genes by DNA synthesis machines; and
- Creating orthogonal biological systems based on a biochemistry not found in nature.

So far most SB scientific papers and conference presentations deal with engineering biological circuits and finding the minimal genome^a^. Less attention has so far been placed on protocells and orthogonal systems; however, some excellent work has been carried out by a couple of very dedicated research groups.^13^–^18^ Protocell research aims to identify ways to produce life out of non-living matter, trying to understand the origin of life and identify new biotech production systems. Researchers working on orthogonal biological systems, on the other hand, try to alter the basic biochemical building blocks of life, such as the nucleic acids or the bases used to encode genetic information.

**Figure 1:**
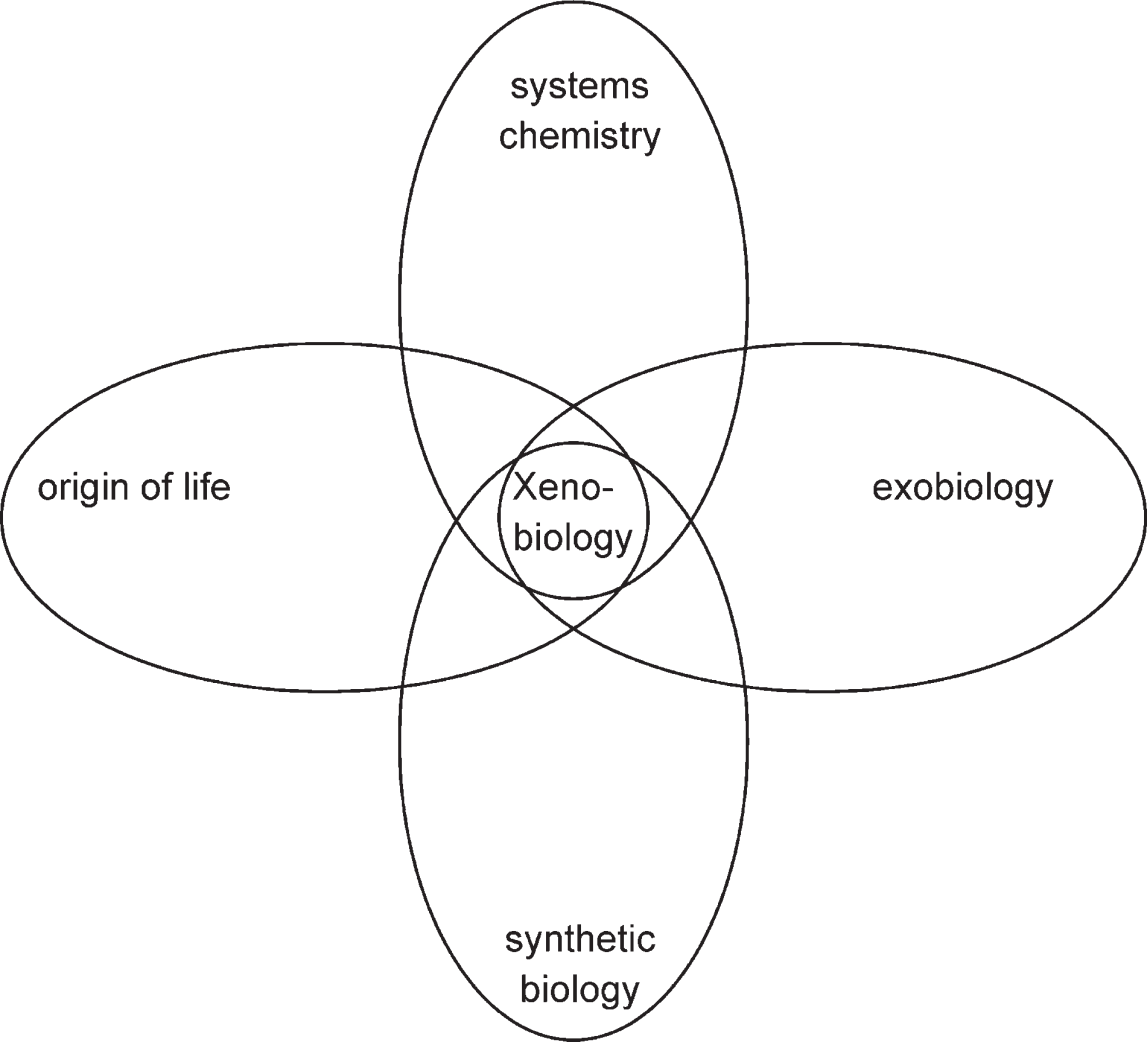
The shared interest in Xenobiology is what the origin of life community, astrobiologists, system chemists, and synthetic biologists have in common.

What the origin of life research community, exobiologists, system chemists and synthetic biologists have in common, is the view that unusual life forms – in other words: xenobiology – could either be found on or beyond Earth, or be deliberately created in the laboratory (Fig. 1). The most obvious difference between them, however, is that the origin of life community and astrobiologists are more interested in “understanding” why life has evolved as it is, while most synthetic biologists are interested in “applying” engineering principles to create unnatural life forms for useful purposes. This paper deals with SB and its attempt to create orthogonal biological systems based on a biochemistry not found in nature.

## Orthogonal life

Ever since industry (*e.g*., mechanical engineering, computer industry) embraced the concept of modularity, it has experienced previously unimaginable levels of innovation and growth. Modularity means building complex products from smaller subsystems that can be designed independently yet function together as a whole. Modularity freed designers to experiment with different approaches, as long as they obeyed the established design rules.^19^ One of the key requirements of modularity, however, is orthogonality. The term orthogonality stems from Greek orthos, “straight,” and gonia, “angle.” The term has originally been used to describe the mathematical situation where two vectors are perpendicular, in other words form a right angle. Changes in the magnitude of one vector do not affect the magnitude of the other vector. In engineering, orthogonality is a system design property facilitating feasibility and simplicity of complex designs. Orthogonality guarantees that modifying one component of a system does not propagate side effects to other components of the system. With the clear benefit of orthogonality in complex systems in mind, synthetic biologists are now trying to apply these engineering principles to biology. However, while engineers have been quite successful applying the principles of orthogonality to the non-living world, biologists still have to overcome major challenges as natural life forms hardly exhibit a true orthogonal design.^20^,^21^ The efforts undertaken by synthetic biologists to construct orthogonal biological systems are two-fold, focusing either on the metabolism or on the biochemical building blocks.

### Metabolic orthogonality

In genetic engineering, the term engineering can only be understood as a metaphor. For example, any recombinant protein that is synthesized in a bacterial cytoplasm can potentially interact with any other cytoplasmic protein, catalyze reactions with any of the several hundreds of metabolites or otherwise interact with any important physiological process.^20^ Therefore, it is almost impossible to design and predict the effect of a new protein in the host cell. One approach in SB is the assembling of a modular platform for the highly efficient synthesis of fine chemicals. The aim is to disentangle the metabolic network (*e.g*., protein-protein interaction) of a cell into particular synthetic modules that do not interact with each other. For example, an energy module and a saccharide production module may be designed with no enzymatic “cross-talk.” Separating the two metabolic modules would allow the productivity of individual modules to be adapted by reengineering its key enzymes without affecting the other module^b^.

### Biochemical orthogonality

Adding another degree of orthogonality, researchers have started to modify and exchange some of the elementary biochemical building blocks of life. The focus of their efforts has been to come up with alternative biomolecules to sustain living processes. Areas of research include the chemical modification of amino acids, proteins or DNA. One area of research is the identification of amino acid sequences (proteins) that have a stable architecture but that do not occur in nature. Actually, only a tiny fraction of proteins that are theoretically possible occur naturally, with many more possible but not-yet-assembled proteins.^16^,^22^,^23^ Other attempts have been made to generate “mirror life,” *i.e*., life that uses molecules that have the opposite chirality of natural life forms.^24^ Changing the translational mechanism from mRNA to proteins *via* tRNA and the ribosome is another focus of interest. For example, *in vivo* incorporation of non-canonical amino acids into proteins in response to an amber nonsense codon has been achieved in *Escherichia coli*, *Saccharomyces cerevisiae* and mammalian cells.^25^–^27^ The triplet codons could thus theoretically code for up to 64 different amino acids. But why stop with 64? First experiments have shown that amino acids can also be encoded in quadruplets, which would theoretically allow for 256 different assignments.^28^ The ability to incorporate more than the 20 canonical amino acids will lead to novel and unnatural proteins and represent an increased diversity of the interpretation of the genetic code.^29^

## Xenobiology

### Expanding the genetic alphabet

Those who believe in the beauty of naturally evolved DNA might be surprised by recent efforts to free-up life (as we know it) from its evolutionary constraints. One can gaze at the biological diversity on our planet and still be stunned about the chemical uniformity of present biological life. SB includes biologists and chemists who are trying to produce unnatural molecules and architectures^3^ in order, eventually, to create xenobiological systems. To come up with an orthogonal chromosome, it is necessary to focus on the nucleotides. The genetic code of all living organisms does not know more than eight nucleoside triphosphates, four in RNA and four in DNA. Synthetic biologists have now altered these canonical nucleotides to the effect that natural biological organisms and systems cannot read and interpret them any more. Experiments replacing or enlarging the genetic alphabet of DNA with unnatural base pairs led for example to a genetic code that instead of four bases ATGC had six bases ATGCPZ.^17^,^30^,^31^ In a recent study, 60 candidate bases (that means 3,600 base pairs) were tested for possible incorporation in the DNA.^18^ These unnatural bases are not recognized by natural polymerases, and one of the challenges is to find/create novel types of polymerases that will be able to read the unnatural constructs. At least on one occasion a modified variant of the HIV-reverse transcriptase was found to be able to PCR-amplify an oligonucleotide containing a third type base pair. Only two amino acids were substituted in the natural polymerase optimized for the four standard nucleotides to create one that supports repeated PCR cycles for the amplification of an expanded genetic system. It is surprising to find a useful polymerase so close in “sequence space” to that of the wild-type polymerase.^17^,^30^,^32^,^33^

### Time for a new backbone: Xeno nucleic acids (XNAs)

**Figure 2:**
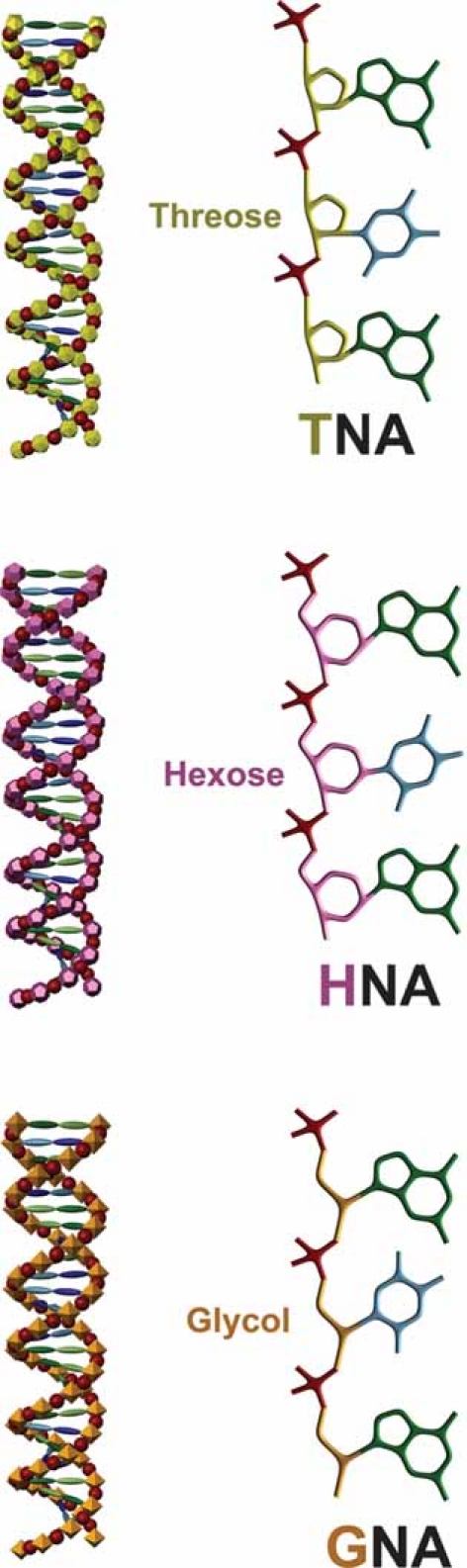
Several xeno nucleotides can form Watson-Crick type double helices. These XNAs can be used as alternative information storing biopolymers. GNA, glycol nucleic acid; TNA, threose nucleic acid; HNA, hexitol nucleic acid (Illustrations by Simone Fuchs).

Another attempt to come up with unnatural nucleotides focuses on the backbone or the outgoing motif of the DNA. Originally this research was driven by the question of how life evolved on earth and why RNA and DNA were selected by (chemical) evolution over other possible nucleic acid structures.^1^ Systematic experimental studies aiming at the diversification of the chemical structure of nucleic acids resulted in completely novel informational biopolymers (see Table 1 and Fig. 2):

**Table 1:** Overview of some xeno nucleotides created so far

Short name	Nucleotide name	Backbone	Base pairs	Outgoing motif	References
HNA	hNTP	Hexose	A-T, G-C	Triphosphate	3,35
TNA	tNTP	Threose	A-T, G-C	Triphosphate	36–38
GNA	gNTP	Glycol	A-T, G-C	Triphosphate	39
CeNA	ceNTP	Cyclohexenyl	A-T, G-C	Triphosphate	37
LNA	lNTP	Ribose with an extra bridge connecting the 2′ and 4′ carbons	A-T, G-C	Triphosphate	40,41
PNA		Desoxyribose	A-T, C-7DG^a^	Protein	42–44

7-Deazaguanine.

Although the genetic information is still stored in the four canonical base pairs, natural DNA polymerases cannot read and duplicate this information. In other words the genetic information stored in XNA is “invisible” and therefore useless to natural DNA-based organisms. To maintain orthogonality it is imperative that no polymerase is available that would convert DNA into XNA, or DNA into XNA, as long as the genetic information is still encoded in the canonical four bases using triplet encoding.

Information storage orthogonality will likely come about through a series of sequential small steps and developments. In a nutshell, the most important challenges to be solved before an XNA-based safe organism can exist *in vivo* are:

- Chemical synthesis of single-stranded XNA
- Auxotrophic biological synthesis of xeno nucleotides, using either
  - Canonical four bases: *x*AMP, *x*GMP, *x*TMP, *x*CMP, or
  - Non-canonical bases
- Defining and biosynthesizing highly specific XNA binding proteins, *e.g*., for
  - Replication: XNA polymerase(s), XNA helicase, XNA ligase, XNA single-strand binding proteins
  - Transcription: defining highly specific transcription factors with an XNA binding domain enabling XNA binding RNA polymerase(s)
  - XNA binding histones to form large-scale genome structures
- Replacing DNA genome with XNA genome
- Possibly removing ATP, CTP, and GTP from cell physiology.

Currently no living organisms based on such an unnatural nucleic acid exist and there is little evidence that anything like it will occur anytime soon. But the combination of an extended genetic code and an adequate novel polymerase could certainly lead to the next step toward implementing an artificial genetic system *in vivo*.^2^,^30^

## The ultimate biosafety tool: A genetic firewall

The road toward the first XNA-based organism will also help philosophers to improve the deductive and inductive reasoning regarding the fundamental question: what is life? When we realize that life does not have to be, and is not always based on, a certain set of biochemical compounds, we will be able to come up with a better concept of life. Future research will most likely expand our concept of life even more, including still more different forms of life, maybe based on silicon instead of carbon, or without the tripartite DNA/XNA-RNA-protein architecture, or without explicit information storing devices altogether.

The potential benefits of orthogonal xenobiological systems might only become relevant over the long term. Over the short term, it is much easier to keep working with DNA than to voluntarily make the already imprecise recombinant DNA work (compared to mechanical engineering) even more challenging.

For the time being the combination of the abilities of the life science R&D together with existing biosafety and biosecurity measures seem to be finely balanced and hardly challenged. With probably millions of recombinant DNA experiments carried out in the past 35 years and post Asilomar biosafety guidelines in place, there is – apart from some infrequent BSL three and four laboratory accidents – no evidence whatsoever to assume that genetically modified organisms have wreaked havoc on our planet or are the source of major pandemics. If everything is fine right now, why develop XNA-based biological systems? Why embark on such a difficult and laborious journey?

**Figure 3:**
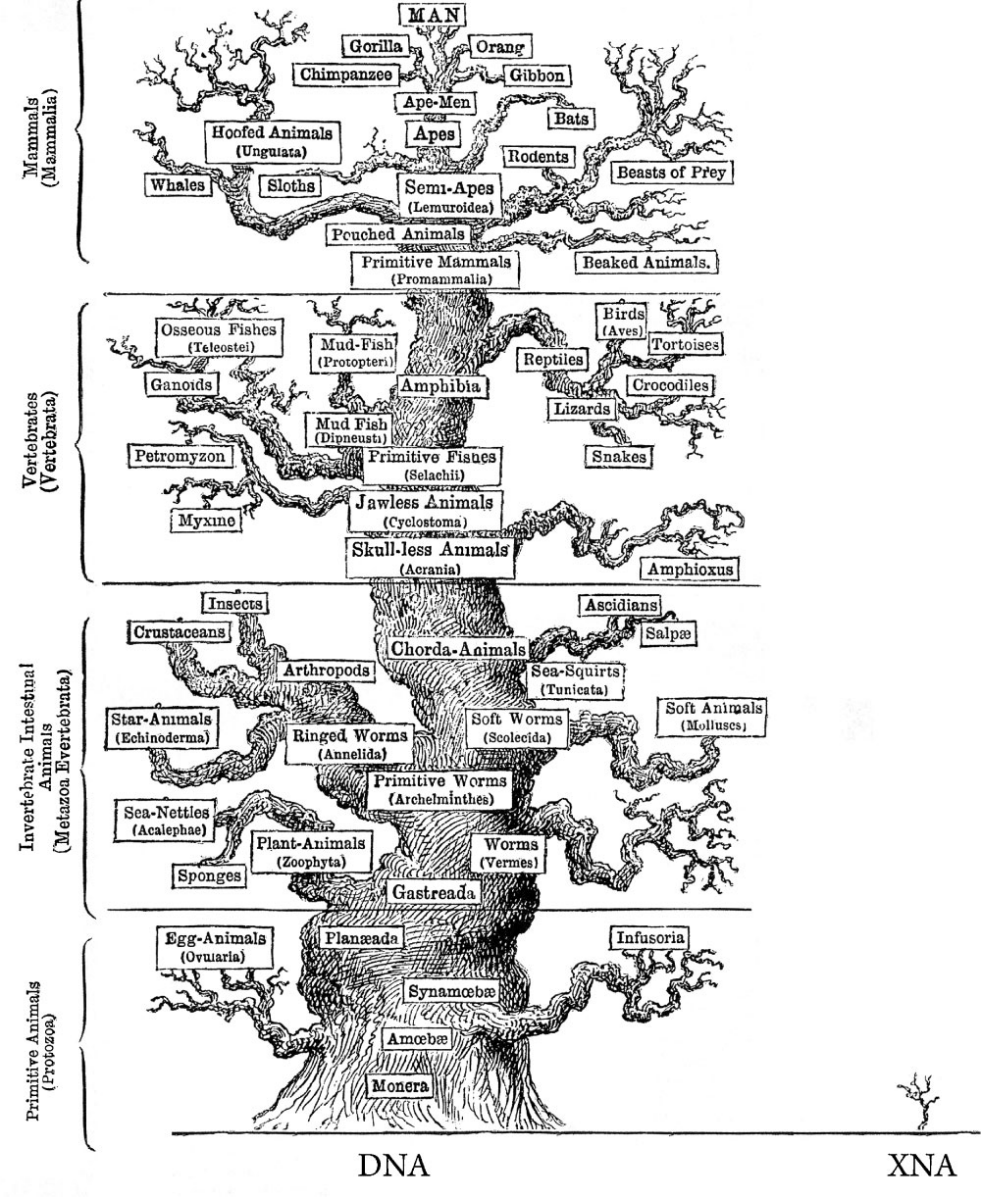
After 4 billion years, a new tree will sprout in the “Garden of Eden”. Non-DNA-based biological systems will be a safer place to conduct SB experiments and applications (38 modified).

Over the medium and long term, it will make sense to design and construct a hardware and software of life that is of different character than the hardware and software of our own life (see Fig. 3). The first 35 years of genetic engineering were just a prelude to what comes in the next 35 years and beyond. From my point of view, the upcoming development in bioengineering will be shaped by the following driving forces:

- Key supporting technologies, such as sequencing and DNA(XNA) synthesis, will become much cheaper and more powerful, a development similar to Moore's law in electronics.^34^
- Design and construction of large biological systems instead of just modifying single genes, will improve not only the speed but also the depth of genetic engineering.
- R&D experiments will soon be carried out by robots, both physically and conceptually, further decreasing the costs and increasing the number of experiments.
- More people (and their robots) will be able to carry out those experiments. Soon the *de facto* monopoly of academia and industry will be gone, giving rise to a new breed of inspired biohackers and amateur biologists.^35^
- Converging or “living technologies” will increasingly bring together hardware, software, and wetware.^36^
- DNA is becoming a molecule of choice also for non-biological applications, *e.g*., as templates for nanotechnology self-assembly systems.^37^
- Potential public fear – and subsequent regulatory red taping – of fast, in-depth and ubiquitous engineering of our own genetic (source) code could stifle further developments and opportunities.

There is little doubt that the amount and complexity (or depth) of DNA-based engineering will not only double or triple over the next decades, but it will increase in the orders of magnitude.

Whatever new or improved physical containment mechanisms are developed, there is one key problem that cannot be solved: all biotech (and nanobiotech) use the same “software program,” namely DNA. DNA occurs in all naturally evolved and domesticated microbes, plants, and animals. Instead of bug fixing, and poorly adjusting biosafety regulations, red taping R&D, or painfully trying to fight off public resistance, why not switch to a different genetic software program altogether? Why not prepare a safe foundation for all the billion and trillion future biotech experiments and applications? Why not switch to another hardware that is incompatible with everything nature has ever created. Why not construct a genetic firewall that solves this problem once and for all?

### Introducing a genetic firewall

Xenobiology could become a fundamental safety device capable of limiting any kind of genetic interaction with the natural world. What xenobiology could bring about is no less than to provide an isolated genetic enclave within the natural world.^39^ In this scenario, xeno-organisms would be able to maintain all basic functions of life such as compartmentalization, metabolism, replication, reproduction, environmental interaction, growth, *etc*. There are, however, some key differences between the xeno and the natural world, and these differences are exactly what makes the genetic firewall so interesting in terms of safety:

- The xeno-organisms must not and cannot produce certain essential biochemical building blocks, *i.e*., their own nucleotides. These biochemicals will have to be supplied externally. Establishing xeno-organisms as a mandatory auxotrophic form of life will allow the limitation of its environmental dispersion by its human creator-designer, providing an extremely tough safety tool. To avoid natural supply of xeno nucleotides, the XNA building blocks should at least be two synthetic steps away from any natural molecule.
- Because natural and xeno-organisms are supposed to use a different and very specific set of nucleotide binding proteins for replication and transcription, gene flow – whether horizontal or *via* sexual reproduction – cannot occur between the two realms of life. DNA cannot be interpreted by the XNA replication machinery and *vice versa*. A piece of XNA cannot, therefore, escape to wild-type organisms and be incorporated into their DNA genomes. Also the XNA organism cannot benefit from genes “discovered” by (natural) evolution through horizontal gene flow (but only through deliberate engineering, and XNA internal evolution). An additional increase in orthogonality and thus safety would be the deployment of several orthogonal systems, such as XNA with different non-canonical bases and rearranged codon assignment.

**Figure 4:**
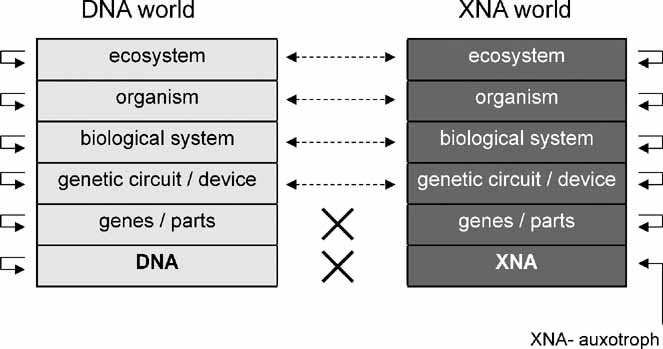
A small step for a molecule, but a big step for safety. The DNA world and the XNA world would be able to interact on the level of whole organisms (*e.g*., providing nutrients, capturing CO_2_, detecting environmental pollutants) but would not able to exchange genetic material through horizontal gene transfer or *via* sexual reproduction. Therefore, it acts as a genetic firewall, but not as a biological firewall. In contrast to the natural world, the XNA world is completely dependent on external supply of essential biochemical building blocks that cannot be synthesized either by XNA or DNA organisms. Any “escape” of a xeno-organism out of the direct control of humans would automatically lead to death.

Although the exchange of genetic information is not possible, other types of interaction would still be feasible. For example, the XNA organisms could produce, sense or dismantle chemical substances under laboratory conditions or in the environment. In theory, it should be possible to let xeno-organisms interact with each other to form their own ecosystem. These ecosystems, however, would be rather limited in size, as all organisms need to be supplied with their essential biochemicals. XNA provides a genetic firewall, but not a biological firewall. That means that XNA organisms might interact with DNA organisms on an ecological level, but never on a genetic level (see Fig. 4). The genetic firewall would not only work between DNA and one XNA (*e.g*., HNA) but also between different XNAs (*e.g*., HNA and TNA, or GNA and PNA).

### XNA specifications

Biosafety mechanisms have been invented and tested in the past. Since the early 1990s auxotrophic systems have been tested; however, none of them were good enough to be put into practice for environmental release.^40^ Of course, the genetic firewall has to be much, much safer than the DNA safety circuits to be considered useful. The ultimate goal would be a safety device with a probability to fail below 10^−40^, which equals approximately the number of cells that ever lived on earth (and never produced a non-DNA non-RNA life form^c^). Of course, 10^−40^ sounds utterly dystopic (and we could never test it in a life time), maybe 10^−20^ is more than enough. The probability also needs to reflect the potential impact, in our case the establishment of an XNA ecosystem in the environment, and how threatening we believe this is. The most important aspect, however, is that the new safety mechanism should be several orders of magnitude safer than any contemporary biosafety mechanism. To ensure the proposed safety improvements the following biological and technical specifications would have to be met:

- Xeno-organisms must not loose their auxotrophic character.
- Natural organisms must also not be able to produce these essential biochemicals, to avoid a symbiotic relationship with XNA.
- Natural DNA polymerase should not be able to transcribe XNA to DNA.
- Natural RNA polymerase should not be able to transcribe XNA to RNA.
- Artificial polymerase must not be able to transcribe DNA to XNA, or otherwise the XNA would have direct access to 4 billion years of evolutionary experience.
- XNA genes be taken up by DNA organisms should not be recognized by natural transcription factors.
- Preferably, single-strand XNA should not interfer with the transcription process in natural cells (like iRNA).
- Symbiogenesis (the merging of two separate organisms to form a single new organism) between XNA and DNA should not take place.
- XNA must not be a recalcitrant chemical, but should act as food for natural organisms after its death/destruction.
- Preferably, additional layers of orthogonality such as non-canonical base pairs, rearranged codon assignment, *etc*. should be used to increase the safety mechanism even further.

### Kick starting XNA systems

**Figure 5:**
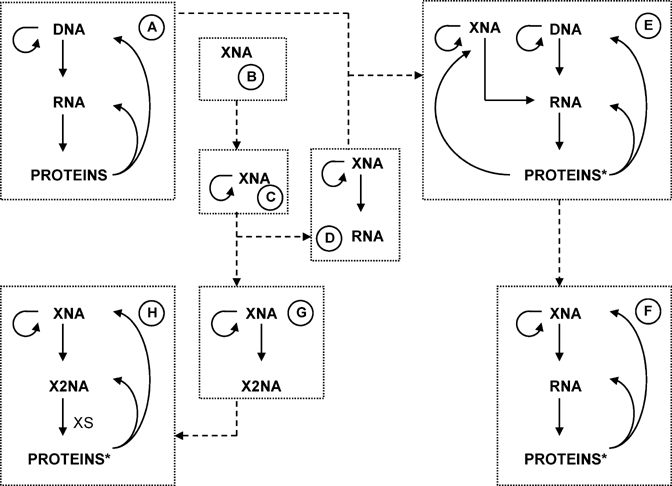
Transition from natural biological architecture to a synthetic architecture is achieved by gradually replacing natural elements with synthetic ones. **A**: Simplified schematic view on the natural replication, transcription and translation system. **B**: In the beginning an XNA biopolymer is a “useless” molecule that lacks cognition in the cell. **C**: XNA-dependent XNA polymerase allow first replication cycles. **D**: Subsequent XNA-dependent RNA polymerase allows the information in the XNA to be transcribed. **E**: Installing this system in a natural cell, both DNA and XNA provide RNA. **F**: Eliminating the DNA from the host cell; the XNA takes over the cell machinery. **G**: Another possible pathway uses XNA-dependent X2NA polymerase, where X2NA means a second type of XNA that is different from the first one (*e.g*., when XNA is HNA, X2NA could be TNA). **H**: To translate X2NA, it will be necessary to modify the ribosome, producing a xenosome (XS) responsible for protein assembly. * Proteins could also be assembled using unnatural amino acids, further enhancing the artificialness of the system. Other applications such as xeno-aptazymes (allosteric xenozymes) are also possible.

To implement a biological system based on XNA, we first need chemically synthesized XNA and an XNA-dependent XNA polymerase for initial replication. The need for specific polymerases is crucial as natural polymerases incorporate unnatural nucleotides rather poorly compared with natural ones.^41^,^42^ Once this has been achieved, we need an XNA-dependent RNA polymerase to transcribe and later translate the genetic information into proteins, using natural ribosomes. Later on, the ribosome could be modified to a xenosome to enable an even higher degree of orthogonality (see Fig. 5).

Since life appeared on earth, natural evolution has – to our knowledge – never produced any XNAs, much less its polymerases. So to get these polymerases we only have two possibilities: design them from scratch, or enhance existing structures to meet our goals. Although we might one day be knowledgeable enough to design it from scratch, the most promising approach right now is directed evolution, where all environmental factors can be controlled. Bacterial organisms or biological subsystems can be rewarded or punished by the operator, potentially leading to XNA replicating systems. Among the most promising approaches in directed evolution is the use of so-called compartmentalized self-replication (CSR). CSR is based on a simple feedback loop within a simple vesicle, in which a polymerase replicates only its own encoding gene. Polymerases that are able to replicate their own encoding gene produce “offspring,” *i.e*., increase their copy number in the post-selection population, while other polymerases that are unable to utilize such primers disappear from the gene pool.^43^,^44^

Based on the concept of whole genome transplantation.^45^,^46^ we would then start with the chemical synthesis of an XNA genome that encodes for its particular polymerases and XNA binding proteins. The XNA genome would be transplanted into a DNA host cell (a minimal genome for example) that is situated in an environment with externally supplied XNA precursors. In the next step XNA polymerase would be added into the host cell to ensure XNA gene expression and stable inheritance of XNA genome to daughter cells alongside the host's DNA. The final step would be the elimination of the host's DNA and complete “takeover” of the cell by the XNA genome and its proteome. By leaving out the last step a dual-NA symbiotic relationship between the two genomes could be imagined as long as both DNA and XNA rely on RNA for transcription. This could potentially jeopardize the genetic firewall of “pure” XNA systems. An additional step toward installing an X2NA system and a xenosome^d^ would solve this bifurcation problem (see Fig. 5).

Still another approach could be possible using protocells. Protocell research aims to create living systems out of non-living chemical materials. Thus, when creating lipid vesicles and adding metabolic ingredients, researchers could implement XNA instead of conventional DNA as the information-storing molecule.^47^ In the future, protocells may even be the basis for different forms of life, *e.g*., one that does not need the tripartite DNA/XNA-RNA/X2NA-ribosome/xenosome structure, but using a completely different chemical architecture.

## How will society deal with a second nature?

When recombinant DNA technology became available to scientists in the 1970s, they were so worried about its potential impact that they organized the now famous Asilomar conference in 1975, to discuss the risks of genetic engineering. Although not all recommendations of Asilomar were put into practice, it was helpful to avoid potential negative consequences of this technology.^48^–^50^ When discussing societal aspects of xenobiology today we need to take the following aspects into account:^51^

- Biosafety: what is the actual probability that XNA life fails on any of the 10 specifications mentioned above? What are the consequences?
- Biosecurity: is there any way XNA could be misused by someone with criminal or malicious intentions? How could it be prevented?
- Intellectual property rights: will the XNA world be owned and controlled by someone, or should it be freely available so anybody could use this safety device? Will some XNAs (*e.g*., TNA) be patented and some (*e.g*., PNA) free?
- Governance: which new rules, guidelines or international treaties need to be established to make sure XNA systems remain as useful as possible? For example, is it necessary to prohibit any activities that actively try to undermine the specifications mentioned above, *i.e*., similar to prohibiting R&D that aims at designing new offensive bioweapons?

In contrast to these rather tangible aspects, we might also be confronted with rather intangible implications. The history of science shows several changes to our worldviews, altering our folk-based narratives to more scientifically inspired (semi-)rational approaches. In this context, science has inflicted a series of disappointments and disillusions to our folk-based beliefs, such as: the earth is not the center of the Universe, men and apes share the same ancestors, or that emotions and thinking is correlated to a neurological substrate. The promoters of these ideas were often attacked by those trying to keep the intellectual status quo. Xenobiology could easily trigger the next paradigm change in the way we understand nature and life. Just as the Earth lost its place as the center of the universe, or men lost its unique status in the animal world, our natural world could lose its unique status as being synonymous with “life.” But as with all other paradigm changes, concepts that better explain the world around us cannot be ignored for long.

## Conclusions

Creation of “alien” or “weird” life in the laboratory, in other words, advances in xenobiology research, will not only contribute to a better understanding of the origin of life, but will definitely expand our capabilities to provide safer biotechnology production tools for human and environmental needs. Future life forms that are orthogonal to natural life forms, such as those based on XNAs, could represent the ultimate biosafety tool. The more layers of orthogonality, however, the safer. A combination of XNA, use of non-canonical base pairs, non-canoncial amino acids, alternative codon assignment, even quadruplet codons, xenosomes, or systems different from the tripartite DNA-RNA-protein architectures will definitely yield orthogonal xenobiological systems that act as genetic firewalls to natural life forms. We should not fear unfamiliar life forms but try to rationally judge their risks and benefits and embrace them in a responsible way for the benefit of humankind.

## Footnotes

a see *e.g*.: SB 2.0: http://webcast.berkeley.edu/event_details.php%3Fwebcastid=15766, SB 3.0: http://www.syntheticbiology3.ethz.ch/, SB 4.0: http://sb4.biobricks.org/

b For an example of this approach see the EUROBIOSYN project description: http://www.eurobiosyn.org/

c Some theoretically possible biological processes never seem to happen in nature. For example, there is no evidence that a DNA organism had itself ever transformed into a HNA, TNA or other XNA organisms. This is so unlikely that it can be considered a biological law, just like the fact that the information of the amino acid sequence of a protein is never be transferred back to the genetic code of another organism. However, there have been many examples where the leading dogma in biology has been challenged. Nobody would have guessed that RNA is transcribed back into DNA, that DNA sequences are heavily rearranged *in vivo*, that pathogens consisting of protein only can propagate, that RNA is catalytic, that acquired properties are inherited under some circumstances. Sometimes “laws” in biology seem rather temporary.

d Xenosome is the synthetic analogon to the ribosome.

## Abbreviations:

SB: synthetic biology
XNA: xeno nucleic acids
